# Dual-targeting triplebody 33-3-19 mediates selective lysis of biphenotypic CD19^+^ CD33^+^ leukemia cells

**DOI:** 10.18632/oncotarget.8022

**Published:** 2016-03-10

**Authors:** Claudia C. Roskopf, Todd A. Braciak, Nadja C. Fenn, Sebastian Kobold, Georg H. Fey, Karl-Peter Hopfner, Fuat S. Oduncu

**Affiliations:** ^1^ Klinikum der Universität München, Medizinische Klinik und Poliklinik IV, Hematology/Oncology, Munich, Germany; ^2^ Ludwig-Maximilians-Universität München, Department of Biochemistry and Gene Center, Munich, Germany; ^3^ Center for Integrated Protein Science (CIPSM) and Klinikum der Universität München, Medizinische Klinik und Poliklinik IV, Division of Clinical Pharmacology, Munich, Germany; ^4^ Friedrich-Alexander-University Erlangen-Nuremberg, Department of Biology, Erlangen, Germany

**Keywords:** leukemia, mixed phenotype acute leukemia (MPAL), immunotherapy, dual-targeting triplebody, selectivity

## Abstract

Simultaneous targeting of multiple tumor-associated antigens (TAAs) in cancer immunotherapy is presumed to enhance tumor cell selectivity and to reduce immune escape.

The combination of B lymphoid marker CD19 and myeloid marker CD33 is exclusively present on biphenotypic B/myeloid leukemia cells. Triplebody 33-3-19 binds specifically to both of these TAAs and activates T cells as immune effectors. Thereby it induces specific lysis of established myeloid (MOLM13, THP-1) and B-lymphoid cell lines (BV173, SEM, Raji, ARH77) as well as of primary patient cells. EC_50_ values range from 3 pM to 2.4 nM. In accordance with our hypothesis, 33-3-19 is able to induce preferential lysis of double- rather than single-positive leukemia cells in a target cell mixture: CD19/CD33 double-positive BV173 cells were eliminated to a significantly greater extent than CD19 single-positive SEM cells (36.6% vs. 20.9% in 3 hours, p = 0.0048) in the presence of both cell lines. In contrast, equivalent elimination efficiencies were observed for both cell lines, when control triplebody 19-3-19 or a mixture of the bispecific single chain variable fragments 19-3 and 33-3 were used. This result highlights the potential of dual-targeting agents for efficient and selective immune-intervention in leukemia patients.

## INTRODUCTION

In acute leukemia of ambiguous origin with B/myeloid or trilineage phenotype (ca. 2 – 3% of all acute leukemias) and B-ALL or AML with aberrant antigen expression, the B lymphoid lineage marker CD19 and myeloid lineage marker CD33 are simultaneously displayed on the blast cell surface [1, 2]. Acute leukemias with co-expression of CD19 and CD33 usually have a poor prognosis [1, 3, 4]. In addition, there is no consensus regarding treatment protocols for mixed phenotype acute leukemias (MPAL) due to the rarity of these hematopoietic neoplasms and lack of clinical studies in this specific patient population [3, 5–7]. However, both CD19 and CD33 are validated therapeutic targets. The co-expression of these two lineage markers may offer unique opportunities for selective, individualized immunotherapy with novel antibody-derived agents, because the leukemia cells are immunophenotypically distinct from the corresponding healthy cells. By targeting both tumor-associated antigens, i.e. CD19 and CD33, at the same time, selectivity of elimination may be achieved and immune escape will likely be reduced, because antigen double-negative leukemia cell clones are less likely to be selected than single-negative ones [8, 9].

A number of multi-specific antibody-derived molecular formats have emerged over the past 25 years [8, 10] including the single chain triplebody platform. Single chain triplebodies are polypeptides that are composed of three single chain variable fragments (scFvs) connected by flexible linkers. They are bi-specific for the target cell and mono-specific for the effector cell [11, 12]. In the classical triplebody format immune effector cells are engaged and activated via the central scFv, which binds to a trigger antigen such as FcγRIII (CD16) on NK cells and macrophages, CD64 or CD89 on neutrophilic granulocytes or the CD3-epsilon chain on T cells. The two distal scFvs in dual-targeting triplebodies bind to two different TAAs on the cancer cell surface. By coupling the recruited immune effector cell to the targeted cancer cell in an Fc receptor- and MHC:peptide-complex-independent manner, the immune effectors are activated and redirected against the target cells [11, 12]. This mode of action - referred to as redirected lysis (RDL) - is also employed by Bispecific T cell Engagers (BiTE®s), including Blinatumomab (BlinCyto™) and AMG330, which target CD19 and CD33, respectively [13–15]. Blinatumomab is the first-in-class of a new group of biotherapeutics after its FDA-approval for the treatment of relapsed and refractory adult B-ALL in December 2014. However, BiTE®s have a number of limitations including their low molecular weight (55 – 60 kD), which results in a serum half-life of only 1.5 to 2 hours [14, 15], and targeting of a single tumor-associated antigen (TAA). Since the latter is not tumor-specific, it does not allow for a very strong discrimination between malignant and normal antigen-positive target cells.

Triplebodies have a molecular mass of 80 to 90 kDa, which is above the threshold of immediate renal clearance. This is reflected in their extended serum half-life of 4 hours in mice in comparison to 2 hours for bispecific single chain Fvs (bsscFvs) [16]. Based on the slower clearance rate compared to bsscFvs, the smaller size compared to monoclonal antibodies and their capacity for multivalent tumor targeting, triplebodies are expected to achieve an efficient penetration of solid tissues [10, 11, 17]. A number of different triplebodies have been developed, which are capable of recruiting NK cells, γδ T cells and T cells as immune effector cell populations for RDL and of targeting a variety of different TAAs that are relevant in hematopoietic malignancies [16, 18–23].

In the present study, we constructed a T cell-recruiting triplebody 33-3-19 to explore the question whether preferential lysis of B/myeloid cancer cells relative to cells expressing only one lineage marker is also possible with the help of T cells as cytolytic effectors. Triplebody 33-3-19 was able to activate resting T cells to induce their proliferation and effector cell activity. Moreover, it enhanced the selective lysis of CD19/CD33 double-positive leukemia cells relative to CD19 single-positive targets with comparable target antigen density, which were present in the same reaction environment. These results lend further support to the concept of enhanced selectivity of lysis mediated by dual-targeting.

## RESULTS

### Construction and properties of dual-targeting T cell-engaging triplebody 33-3-19

**Figure 1:**
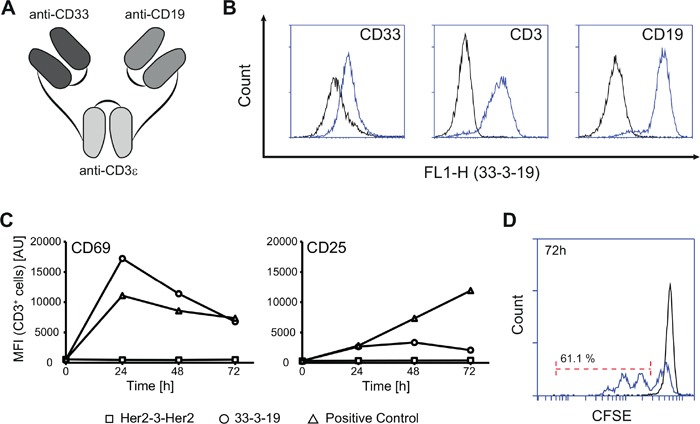
Specific binding and T cell-activation induced by dual-targeting triplebody 33-3-19 **A.** Design principle of 33-3-19. **B.** Specific binding of 33-3-19 to its target antigens as determined by cytofluorimetry. The antigen-positive targets used were the MOLM-13 (CD33) and SEM (CD19) cell lines and primary T cells (CD3) isolated from a healthy donor. No binding was observed to antigen-negative cells (HEK 293, data not shown). **C.** Non-stimulated PBMCs were incubated at an E : T ratio of 1 : 2 with SEM target cells and 1 nM triplebody or 2% PHA/100 U/mL IL-2 (pos. control) for 72 hours. The expression of activation markers CD69 and CD25 by effector T cells was assessed (n = 4). Representative data from one 28-yr old, healthy, male donor (70.4% CD3^+^, 4.5% CD19^+^) are shown. At time t_0_ the overall content of CD19-positive cells in the reaction mixture (PBMCs + SEMs) was 14.6%. **D.** Non-stimulated PBMCs were labelled with 5 μM CFSE prior to incubation with SEM target cells at an E : T ratio of 1 : 2 and a cell density of 3 * 10^5^ / mL at t_0_. T cell proliferation was assessed based on dilution of the CFSE cell proliferation dye (n = 3). Black = reaction without triplebody; blue = reaction with 1 nM 33-3-19.

To clone the triplebody 33-3-19 (Figure 1A), the N-terminal anti-CD19 single chain variable fragment (scFv), which was encoded at the 5′-end of the 19-3-19 gene in a pSecTag2-HygroC mammalian expression vector [21], was replaced by a humanized anti-CD33 scFv. Triplebody 33-3-19 and control proteins were purified from the supernatant of stably transfected Freestyle 293F cell pools by Ni-NTA affinity (Figure S1A) and size exclusion (Figure S1B) chromatography. Between 0.5 to 1.5 mg monomeric triplebody were obtained on average from 1 L of culture supernatant. As can be inferred from the size exclusion chromatogram (Figure S1B), triplebody 33-3-19 displayed a marked aggregation tendency. The protein's thermostability was measured by thermal shift assays and two melting points at 58.5°C (CD19 and CD3 scFvs) and at 70°C (CD33 scFv) were recorded. The biological activity of 33-3-19, which was determined from its EC_50_-value in a standard redirected lysis (RDL) experiment against SEM target cells at different time points post production (data not shown), weakened over time, in spite of stabilization attempts via a variety of formulation-buffers, disulfide stabilization and site-directed mutagenesis (data not shown). However, triplebody 33-3-19 was suitable for proof-of-concept studies as it bound specifically to its three target antigens CD33, CD3-epsilon and CD19 (Figure 1B) and displayed a strong biological activity when used within one to two months after production.

### Activation of resting T cells by triplebody 33-3-19

We first tested the ability of 33-3-19 to activate resting T cells. 33-3-19 was added to cytolysis reactions of unstimulated mononuclear cells (MNCs) from healthy donors, which were mixed with SEM (CD19-positive pre-B ALL cell line) target cells at an effector-to-target (E : T) ratio of 1 : 2. Within 24 to 48 hours after treatment with 33-3-19, the expression of early activation marker CD69 on the surface of the CD3^+^ cell population was strongly increased (Figure 1C). Expression of CD25, the alpha-chain of the IL-2 receptor, on the T cell surface was also increased (Figure 1C) with different kinetics than CD69. The response patterns for the expression of activation markers varied considerably between different blood donors, but upon reaching peak cytolysis levels of 94.2 - 99.6 % of target cells after 48 hours, the levels of both CD69 and CD25 on the T cell surface began to drop again in each case. Addition of triplebody 33-3-19 to T cells without antigen-positive target cells did not lead to the elevation of activation marker levels (data not shown), nor did the addition of a Her2-3-Her2 control triplebody to a reaction mixture containing T lymphocytes and leukemia cells (Figure 1C). This result suggests that T cell activation was not due to HLA-mismatch between donor and leukemia cells. Further, the presence of target antigen, which was physically linked to the surface of the T cell via the mediator protein, was thus an essential requirement for triplebody-mediated activation of T lymphocytes.

In parallel to the elevation of activation markers on the T cell surface, a donor-dependent elevation of the concentration of cytokines IL-2, IL-6, IL-10, TNFα and IFN-γ in the supernatant was detected (Figure S2A). To further analyze the impact of 33-3-19 on T cell proliferation, MNCs were fluorescently labelled with the cell proliferation dye CellTrace™ CFSE prior to the cytolysis reaction. More than 60 % of CD3^+^ cells had already run through 1 to 3 cell cycles after 72 hours (Figure 1D). After 120 hours more than 97 % of T cells had proliferated (Figure S2B). No cytokine release or T cell proliferation was observed in control reactions without triplebody or with the specificity-control triplebody Her2-3-Her2.

### Efficient redirected lysis of ALL and AML target cell lines

**Figure 2:**
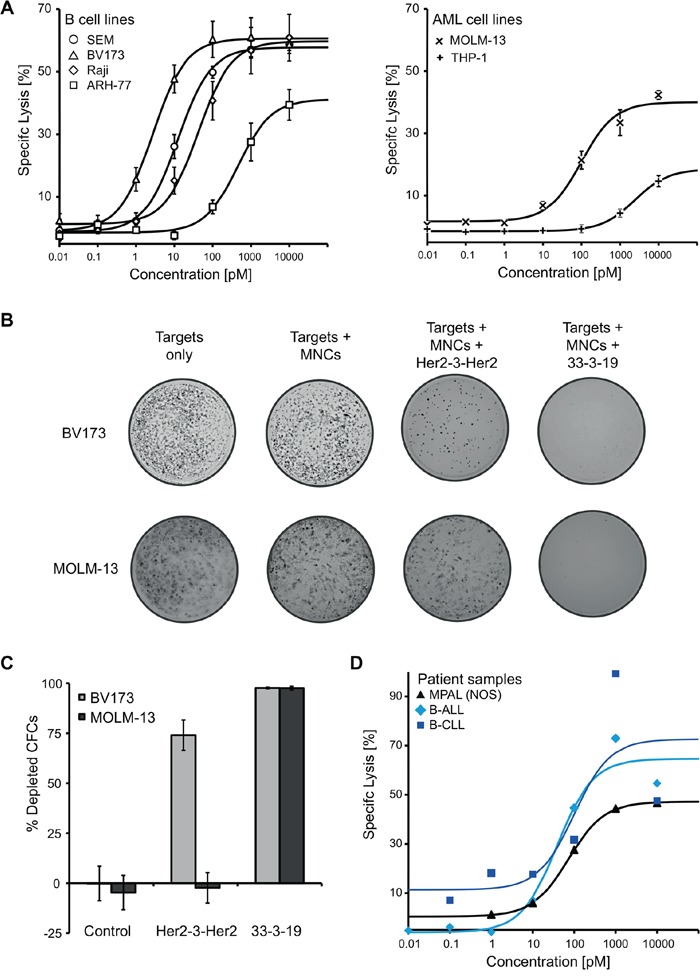
33-3-19-mediated lysis of B and AML cell lines including their colony forming cells (CFCs), as well as of primary patient material **A.** Dose-response of several B-lymphoid (left) and AML cell lines (right) representing different types of hematologic malignancies. No cytolytic response was observed, when the specificity-control triplebody Her2-3-Her2 was employed (data not shown). **B + C.** Cells were harvested post cytolysis and used in a human colony-forming cell (CFC) assay. 5.5 * 10^4^ (MOLM-13 targets) or 1.1 * 10^5^ cells (BV173 targets) were seeded into each well, respectively, which corresponds to 5,000 seeded MOLM-13 and 10,000 seeded BV173 cells (the remaining cells are the MNC effector cells). After 7 days, cells were stained with 1 mg/mL iodonitrotetrazolium-chloride solution overnight. Images were taken on the following day and colonies counted manually (n = 3 for each cell line). **D.** Dose-response of primary patient material (PBMCs) to treatment with triplebody 33-3-19 plus allogeneic PBMCs. All patient samples were collected at first diagnosis. The MPAL (NOS) patient displayed a trilineage phenotype (B lineage: CD19^high^, CD79a^high^; T lineage: cyCD3^+^, CD2^+^, CD5^high^, CD7^high^; myeloid lineage: MPO detectable, CD33^+^, CD117^high^).

To determine the efficiency of 33-3-19-induced, T cell-mediated cytolysis of target antigen-positive cells, redirected lysis assays were performed with different AML and B cell lines as targets and with *ex vivo* expanded, pre-stimulated, allogeneic MNCs as effectors. An effector-to-target-cell ratio of 10 : 1 and an incubation time of 3 hours were employed. The expression of either CD19 or CD33 on the cancer cell surface was sufficient to induce cytolysis via 33-3-19 plus T cells (Figure 2A). However, cytolysis was not induced in the absence of target antigen on the cancer cells as determined with the specificity control Her2-3-Her2 (data not shown). The extent of cytolysis was concentration-dependent and a trend towards higher maximum lysis and lower EC_50_-values was observed with higher target antigen density on the cell surface (Table 1). EC_50_-values for the B lymphoid cell lines were in the low picomolar range (3 – 460 pM). The tested AML-cell lines responded at higher triplebody concentrations with EC_50_-values of 0.1 nM (MOLM-13) and 2.4 nM (THP-1), respectively (Table 1).

**Table 1:** EC_50_-values, maximum specific lysis and antigen density for 33-3-19-sensitive cell lines and patient samples

		Disease	EC_50_ (95% CI) [pM]	Max. spec. lysis [%]	Antigen Density [molecules/cell]	
					CD19	CD33
**B cell lines**	**SEM**	*Pre-B ALL*	12 (8 – 18)	58.7	50,000 ± 17,000	< 100
	**BV173**	*Pre-B ALL*	3 (1 – 6)	61.0	60,000 ± 11,000	4,500 ± 800
	**Raji**	*Burkitt's lymphoma*	42 (18 – 98)	60.8	31,000 ± 19,000	650 ± 100
	**ARH77**	*Plasma cell leukemia*	460 (179 – 1,177)	39.4	3,000 ± 2,000	< 100
**AML cell lines**	**MOLM-13**	*AML-M5a*	100 (58 – 173)	42.3	0	32,000 ± 6,500
	**THP-1**	*AML-M5*	2,442 (1,105 – 5,311)	14.6	0	17,000 ± 4,000
**Patient samples**		*ALL*	39 (9 – 165)	72.9	n.d	n.d.
		*MPAL(NOS)*	72 (63 – 84)	46.6	8,500 ± 3,000	300
		*B-CLL*	101 (1 – 44,550)	99.2	n.d.	n.d.

EC_50_-values and maximum specific lysis were determined from the sigmoidal dose-response in 3 hour Calcein release cytolysis assays with an E : T Ratio of 10 : 1. Antigen density was determined by calibrated cytofluorimetry using the QifiKit (Dako) [28]. N.d. = not determined.

### Elimination of potential leukemia-initiating cells

To achieve long-lasting remissions, it is necessary to eliminate those cancer cells that are capable of repopulating the cancer tissue, i.e. the leukemia-initiating cells (LICs) and especially the leukemia stem cells (LSCs). One hallmark of LICs is their colony-forming capacity. To investigate whether treatment with 33-3-19 leads to the eradication of LICs as well as bulk cancer cells, we performed colony forming cell (CFC) assays with the cells that had survived a 4 hour redirected lysis assay with or without triplebody. The addition of 33-3-19 resulted in the elimination of more than 97% of CFCs from a biphenotypic Philadelphia chromosome-positive B-precursor ALL cell line (BV173) as well as a CD33^+^ AML M5a cell line (MOLM-13) (Figure 2B and 2C). This result points towards the capacity of triplebodies to eradicate potential LICs and warrants further careful examination in the future with primary patient cells as targets.

### Redirected lysis of primary material from patients with different disease entities

To determine, whether triplebody 33-3-19 was also effective against primary cancer cells, redirected lysis assays were performed with primary cells from three patients, diagnosed with B-CLL, B-ALL and mixed phenotype acute leukemia (no other specification) (MPAL (NOS)), respectively. Each leukemia cell sample responded to treatment with 33-3-19 plus allogeneic effector cells in a dose-dependent manner, and maximum specific lysis values of 46.6% (MPAL (NOS)), 72.9% (B-ALL) and 99.2% (B-CLL) were achieved within 3 hours (Figure 2D, Table 1). EC_50_-values ranged from 40 to 100 pM. The blasts from the patient with MPAL (NOS) displayed a combined (CD19 plus CD33) target antigen density of approximately 9,000 molecules/cell (Table 1). This - together with its maximum lysis and EC_50_-value - supports the notion that combined target antigen density correlates with higher maximum specific lysis/lower EC_50_-value. In the samples from the B-CLL and B-ALL patient a higher degree of specific lysis was achieved with 1 nM than with 10 nM triplebody (Figure 2D).

### Enhanced selectivity of lysis for biphenotypic CD19^+^ CD33^+^ target cells

To assess whether the dual-targeting of CD19 and CD33 with a single molecule actually enhanced the selectivity of target cell lysis in a mixed environment, cytolysis experiments with mixed target cell populations were performed. The target cell population was composed of a mixture of CD19 single-positive SEM cells and CD19/CD33 double-positive BV173 cells. The SEM cell line was chosen, because of its comparably high target antigen density: SEM cells carried approximately 50,000 CD19 molecules and no detectable CD33 molecules on their surface, BV173 cells carried approximately 60,000 copies of CD19 and 4,500 copies of CD33 on their surface (Table 1).

**Figure 3:**
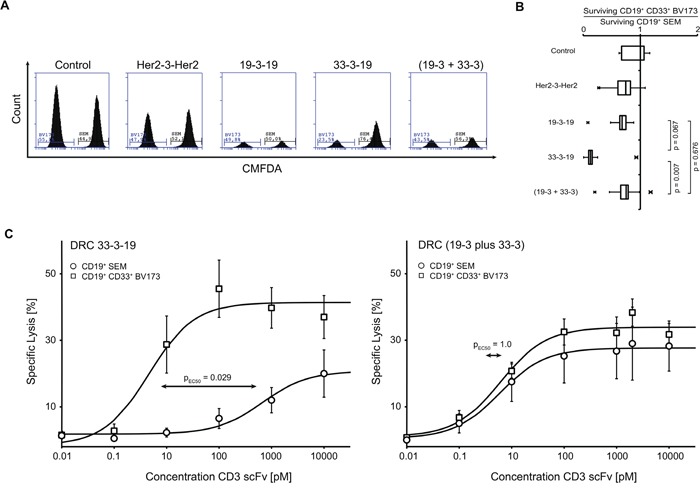
Selective lysis of CD19/CD33 double-positive target cells induced by dual-targeting triplebody 33-3-19 plus allogeneic T cells Cytolysis assays with mixed CD19 single-positive (sp) and CD19/CD33 double-positive (dp) target cell populations were performed to investigate whether dual-targeting triplebody 33-3-19 achieved selectivity of lysis. **A.** PBMCs were labelled with 2 μM CellTracker™ Deep Red and sp SEM and dp BV173 target cells were labelled with 2 μM and 20 nM CellTracker™ Green CMFDA, respectively, and mixed at an E : sp T : dp T ratio of 1 : 3 : 3. After incubation for 12 hours cells were stained with 7-AAD to exclude dead/dying cells and the number of surviving sp and dp target cells was assessed by flow cytometry (n = 5). Figure 3A shows an example of the histograms for surviving CMFDA^+^ 7-AAD^−^ Deep Red^−^ target cells from each reaction. **B.** The ratio of surviving dp BV173 to sp SEM target cells was calculated from absolute cell numbers in a set sample volume (250 μL). The box plot in Figure 3B is based on five independent measurements. On average, three-times more sp SEM target cells survived treatment with 1 nM dual-targeting triplebody 33-3-19 than dp BV173 target cells. Treatment with 1 nM mono-targeting triplebody 19-3-19 or an equimolar concentration of bsscFvs (19-3 plus 33-3) reduced both target cell lines to a similar extent. **C.** sp SEM target cells were Calcein-labelled in one and dp BV173 target cells were labelled in the other arm of a 3 hour Calcein release cytolysis assay with mixed target cell populations (E : sp T : dp T is 2 : 1 : 1). The concentrations of dual-targeting triplebody 33-3-19 and an equimolar mixture of the CD3 bsscFvs (19-3 plus 33-3) were titrated. The ratio of killed sp-to-dp target cells was determined from the ratio of specific lysis achieved in each arm of the experiment, respectively (n = 4). While both cell lines were equally sensitive towards treatment with the bsscFv mixture (EC_50_-values are 6.1 pM for BV173 and 5.9 pM for SEM), the double-positive cell line BV173 was 145-times more sensitive towards treatment with 33-3-19 (EC_50_-value = 4.6 pM) than the single-positive cell line SEM (EC_50_-value = 667.7 pM), when both target cell lines were present in the same reaction volume.

In a first approach, the target cell populations were labelled with different concentrations of a permanent fluorescent dye and mixed with pre-stimulated MNCs at an E : T ratio of 1 : 6. After 12 hours the surviving target cells were enumerated cytofluorimetrically. Upon addition of 1 nM dual-targeting triplebody 33-3-19, the double-positive BV173 target cells were lysed preferentially over the single-positive SEM target cells, as demonstrated by a three-fold higher viability of the SEM cells (Figure 3A). In contrast, the monospecific bivalent triplebody 19-3-19 or a mixture of the bsscFvs (19-3 plus 33-3) reduced both populations to a similar extent: treatment with 33-3-19 resulted in a ratio of 0.13 for surviving BV173-to-SEM target cells (Figure 3B), while treatment with the bsscFv mixture (19-3 plus 33-3) resulted in a significantly different ratio of 0.66 (t-test, p = 0.007). Treatment with 19-3-19 resulted in a ratio of 0.68 for surviving BV173-to-SEM target cells (t-test, p = 0.067).

To confirm these results by a complementary method, we now labeled each cell type independently with Calcein green in a cytolysis test to determine specific lysis of each target cell population individually (Figure S3A). Again, the BV173 population was lysed to a significantly greater extent than the SEM population upon treatment with 33-3-19, but not after treatment with 19-3-19 or an equimolar mixture of (19-3 plus 33-3) (Figure S3B). Dose-response curves were established for each population in the target cell mixture treated with either 33-3-19 or the bsscFv mixture (19-3 plus 33-3). The concentration of the CD3-epsilon scFv was equimolar between the treatment groups, i.e. 1 nM 33-3-19 corresponded to (0.5 nM 19-3 plus 0.5 nM 33-3). CD19^+^ CD33^+^ BV173 target cells responded to far lower doses of dual-targeting triplebody 33-3-19 than the CD19^+^ SEM target cells, when both were simultaneously present in the reaction mixture (Figure 3C, left panel). The EC_50_-values of 33-3-19 for the double- and single-positive cell lines were 4.6 pM and 667.7 pM, respectively. This is a significant 145-fold difference in sensitivity (Mann-Whitney u-test, p = 0.029) towards 33-3-19 treatment between the two target cell populations. In contrast both, the double- and the single-positive cell lines, displayed equal sensitivity towards treatment with the mixture of bsscFvs (19-3 plus 33-3) with EC_50_-values of 6.1 and 5.9 pM, respectively (Figure 3C, right panel).

## DISCUSSION

In the present study, we characterized a dual-targeting T cell-recruiting triplebody 33-3-19 that was designed for the selective lysis of CD19/CD33 double-positive B/myeloid leukemia cells over CD19 single-positive normal cells. The results emphasize that dual-targeting agents have the capacity to achieve better target cell selectivity and reduced off-target toxicity.

To activate CTLs, to redirect their effector cell functions, and to induce T cell proliferation, the simultaneous binding of the CD3-epsilon trigger antigen on the T lymphocytes and tumor-associated antigen (TAA) on the cancer cell surface by triplebody 33-3-19 was required. This observation as well as our previous experiences with the mono-targeting triplebody 19-3-19 [21] suggest that the mode of action of T cell-recruiting triplebodies is very similar to that postulated for BiTE®s [24–26]: the higher affinity of these antibody derivatives for the TAAs rather than the effector cell antigen leads to a preferential coating of the cancer cells. Once a T cell has come into proximity of the target cell, multiple specific binding events to the triplebodies coating the cancer cell result in CD3-receptor cross-linking and subsequent T cell activation. However, in contrast to BiTE®s, antibody derivatives with multiple targeting domains such as 33-3-19 bind bi-(or multi-)valently to the target cell and can thus profit from a cooperativity or avidity effect, which has previously been demonstrated for several triplebodies [16, 19–21]. Therefore the difference in affinity for the TAA and trigger antigens may not need to be as pronounced as for BiTE® molecules [25]. This may also give rise to lower off-target toxicity, because the affinity for single-positive non-target cells may be too low to induce a prolonged contact with CTLs or other immune effector cells.

Since the mode of action of BiTE®s and T cell-recruiting triplebodies appears to have common components, it is not surprising that the release of cytokines IL-2, IL-6, IL-10, TNFα and IFN-γ was observed after treatment of target cells *in vitro* with 33-3-19 and effector T cells. This result leads to the prediction that T cell-engaging triplebodies may also induce a cytokine release syndrome (CRS) similar to the one described clinically for Blinatumomab [14, 15]. However, the clinical experience with this T cell-activating agent and with the use of (CAR) T cells for therapy have helped to implement CRS treatment strategies, which are effective in most cases [27].

In this study, we also provided clear evidence suggesting that dual-targeting of (CD19 plus CD33) improved target cell selectivity, in particular at sub-saturating concentrations. The presence of only one of the TAAs on the target cell surface was sufficient to redirect T cell function; however, CD19/CD33 double-positive target cells displayed a 145-fold greater sensitivity towards treatment with 33-3-19 than CD19 single-positive cells, when both populations were present in the same reaction environment. This observation points to a possible concentration-dependent therapeutic window for the selectivity of dual-targeting agents: at concentrations of the agent, which fall into this window, double-positive cancer cells are largely eradicated, but single-positive cells are mostly spared. It may be possible to maximize this “selectivity window” by affinity engineering of the individual arms of dual-targeting agents as was recently shown by Mazor *et al.* for an anti-CD4/CD70 DuetMab® [9].

Another important parameter is the combined and individual target antigen density on the target and non-target tissues. The antigen density limits the number of triplebody molecules that can be bound to the cancer cell surface. We frequently observe - for example in the case of the B-CLL and B-ALL patient samples in this study - that target cells display a higher specific lysis at 1 nM triplebody than at 10 nM triplebody. This pattern might be attributed to an “oversaturation” effect: when the amount of triplebody exceeds the number of available binding sites on the cancer cells, the effector cells may be coated in addition to the targets and a successful interaction between effectors and targets to form a cytolytic synapse is mediated less frequently or less efficiently. Thus, the most effective concentration of a dual-targeting agent such as 33-3-19 may depend on the combined target antigen density of an individual patient's target cells. This may pose a challenge for the determination of an appropriate dosing regimen: target antigen densities vary significantly between individuals [28] and can even vary between the bulk of leukemia cells and the leukemia-initiating stem- or progenitor cells (as is the case for the combined density of CD33 and CD123 on AML LSCs [29], for example). Thus, the suitable concentration to achieve a selective eradication of target cells may also vary between patients.

Triplebody 33-3-19 has not yet undergone late-stage preclinical development. Most of the limiting protein-chemical properties described above and in particular the tendency to form aggregates can probably be attributed to intra- and intermolecular shuffling of V-chain subdomains, because the individual scFvs used here were stable in other triplebody constructs. However, recent advances in antibody engineering [30] offer the opportunity to improve the intrinsic stability of antibody derivatives such as 33-3-19. If such stability engineering were combined with affinity engineering to further increase the “selectivity window”, then later-stage versions of 33-3-19 may become potent and clinically useful therapeutic agents for a selective targeting of CD19^+^ CD33^+^ B/myeloid leukemia cells.

## MATERIALS AND METHODS

### Cloning, production and purification

Triplebody 33-3-19 and bsscFv 33-3 were constructed using standard molecular biology techniques. Briefly, the 5′ (i.e. N-terminal) CD19-scFv in our previously described 19-3-19 and 19-3 constructs [21] were replaced with the humanized CD33-specific scFv, which was isolated by polymerase chain reaction (PCR) from the SPM-2 gene cassette [22]. All triplebody- and bsscFv-encoding genes were cloned into the pSecTag2-HygroC vector for mammalian expression. Freestyle 293F cells were transfected with TransIT-LT1 transfection reagent and a stable production cell pool was generated by Hygromycin B selection for 8 weeks. The recombinant protein was purified from the supernatant via Ni-NTA affinity chromatography followed by analytical size exclusion chromatography. Protein aliquots were stored at −80°C.

### Thermal shift assay

SYPRO Orange (Thermo Fisher Scientific, Darmstadt), which only fluoresces upon binding to denatured protein, was added to 45 μL aliquots of a 0.1 mg/mL protein solution at a dilution of 1 : 5,000. Fluorescence emitted by the labeled denatured triplebody was monitored on a Biorad CFX 96 instrument during a temperature increase from 10°C to 95°C at 0.5°C intervals (10 sec/interval).

### Cell culture methods

Cell lines BV173, Raji, ARH77, THP-1 and MOLM-13 were purchased from the German Collection of Microorganisms and Cell Lines (Leibniz-Institut DSMZ, Braunschweig). The SEM cell line was purchased from the American Type Culture Collection (ATCC). SEM, Raji, ARH77 and THP-1 were cultured in RPMI 1640 (Gibco, Thermo Fisher Scientific, Darmstadt) supplemented with 10% fetal bovine serum (FBS) and Penicillin (100 U/mL) / Streptomycin (100 μg/mL). Medium for BV173 and MOLM-13 was supplemented with 20% FBS. Freestyle 293F cells were purchased from Life Technologies and grown in FreeStyle™ medium in a shaking incubator.

Blood samples from healthy donors and patients with hematologic malignancies were drawn into EDTA-monovettes (Sarstedt) after informed written consent had been obtained. This study is in compliance with the declaration of Helsinki and was approved by the ethics committee of the Medical Faculty of the Ludwig-Maximilians-Universität München (project no. 173-13). PBMCs were separated by density gradient centrifugation using Lymphoprep™ (Axis Shield PoC) medium, and residual erythrocytes were lysed by incubation with erythrocyte-lysis-buffer (University Pharmacy, Munich) for 5 minutes. To generate effector cells for standard 3 hours cytotoxicity assays an *ex vivo* expansion and stimulation of mononuclear cells (MNCs) was carried out for 20 days in the presence of IL-2 as described [23, 31]. For T cell activation and proliferation assays freshly isolated, non-stimulated PBMCs were used.

### Flow cytometry

Flow cytometric analyses were carried out on a BD Accuri C6 or a Millipore Guava instrument. For binding studies target antigen-positive cells were incubated with 15 μg/mL antibody derivatives and washed. Bound antibody derivatives were then detected with an AlexaFluor488-conjugated anti-His_5_ antibody (1 : 200 dilution, Qiagen). CD3, CD4, CD8, CD16, CD19, CD25, CD33, CD56, CD69 and isotype control antibodies conjugated with different fluorophores were purchased from Immunotech (Beckmann-Coulter, Marseille). For cell-surface marker analysis, target cells were fixed in 3.7 % paraformaldehyde (PFA) solution at room temperature for 15 minutes, subsequently stained with the required antibody cocktail at 4°C for 30 minutes, washed with phosphate buffered saline (PBS) and analyzed. The viability dye 7-AAD and the Cytometric Bead Array™ Human Th1/Th2 Cytokine Kit II were purchased from BD Biosciences (San Diego, CA) and used according to the manufacturer's instructions. The CellTrace™ CFSE proliferation dye (Molecular Probes, Darmstadt) and the QifiKit (Dako, Eching) for the quantification of surface antigens were used according to the manufacturer's instructions.

### Calcein release assay/redirected lysis assay (RDL)

Target cells were labelled with 15 μM Calcein Green AM (Molecular Probes, Darmstadt), washed, and mixed with *ex vivo* expanded and stimulated PBMCs from healthy unrelated donors in RPMI 1640 GlutaMAX™ supplemented with 10 % FBS and Penicillin (100 U/mL)/Streptomycin (100 μg/mL) (Gibco, Thermo Fisher Scientific, Darmstadt) at an E : T ratio of 10 : 1. Antibody derivatives were diluted to the desired concentration with medium and added to 200 μL reaction volumes in a 96-well round bottom tissue culture plate (CellStar, Greiner bio-one). After a 3 hour incubation period at 37°C, 5 % CO_2_, 100 μL supernatant was transferred to a black 96-well flat bottom plate (Nunc) and fluorescence was determined on a Berthold Mithras plate reader (Berthold Technologies, Bad Wildbad). Maximum lysis was achieved by addition of 2.5 % Triton X-100. Specific lysis was calculated as follows:

% Specific Lysis = 100 * [( RLU (sample) – RLU (background)) / (RLU (max. lysis) – RLU (background))], where RLU = relative light units and the background is the degree of lysis obtained with effector cells alone in the absence of added triplebody.

### Selective lysis studied by flow cytometry

To investigate whether 33-3-19 induces a selective lysis of target antigen double-positive (dp) cells, single-positive (sp) CD19^+^ SEM cells and dp CD19^+^ CD33^+^ BV173 cells were differentially labeled with CellTracker™ Green CMFDA (i.e. 2 μM and 20 nM, respectively) and mixed with *ex vivo* expanded and stimulated, CellTracker™ Deep Red (2 μM)-labelled PBMCs at an E : sp T : dp T Ratio of 1 : 3 : 3. Triplebodies 19-3-19, 33-3-19, Her2-3-Her2 or a mixture of the bsscFvs (19-3 plus 33-3) were added to give 1 nM concentration. After a 12 hour incubation period at 37°C/5 % CO_2_ cells were harvested, stained with the viability dye 7-AAD and resuspended in 500 μL PBS. 250 μL of each sample were analyzed by flow cytometry. Surviving target cells were identified by gating on CMFDA^+^, Deep Red^−^, 7-AAD^−^ cells.

### Selective lysis studied by Calcein release

In an alternative approach to investigate the hypothesized target cell selectivity of 33-3-19, cell death of sp and dp target cells in a mixed population was measured by Calcein release in parallel reactions. In one reaction mixture sp SEM cells were labeled and in the parallel reaction dp BV173 cells were labeled with 15 μM Calcein Green AM. Cell death was determined by fluorescence from released Calcein Green in the supernatant after an incubation period of 3 hours with pre-stimulated PBMCs from healthy unrelated donors at an E : sp T : dp T ratio of 2 : 1 : 1. Triplebodies Her2-3-Her2, 19-3-19, 33-3-19 and the mixture of bsscFvs (19-3 plus 33-3) were adjusted to the desired concentrations. Maximum lysis was achieved by addition of 2.5 % Triton X-100 and specific lysis was calculated as described above.

### Colony formation assays

Colony formation assays in MethoCult™ were performed after a 4 hour cytolysis reaction with an E : T ratio of 10 : 1 PBMCs to target cells and 1 nM triplebody Her2-3-Her2 or 33-3-19. Colony Forming Cells (CFCs) were detected and counted using the MethoCult™ H4434 Classic medium (Stem Cell Technologies, Munich). Briefly, cells were harvested and washed with Iscove's Modified Dulbecco's Medium after the cytolysis reaction. 5,000 to 10,000 target cells were seeded to 1 mL of rigorously vortexed MethoCult™ medium and transferred to a 24-well tissue culture plate. The sample well was surrounded with water-containing wells and the dish was incubated at 37°C/5 % CO_2_ for 7 days. On day seven, 100 μL of a 1 mg/mL iodonitrotetrazolium chloride (INT) solution in PBS was added and after an overnight incubation at 37°C/5 % CO_2_ the number of colonies was counted.

### Statistical analysis

All statistical analyses were performed by GraphPad Prism Software (GraphPad Software Inc., San Diego, CA, USA) using Student's t-test for the determination of significance in normally distributed, and using the Mann-Whitney u test in samples with unknown distribution. Statistical significance was defined as p < 0.05.

## SUPPLEMENTARY FIGURES



## Abbreviations

ALL: Acute Lymphoid Leukemia
AML: Acute Myeloid Leukemia
bsscFv: bispecific scFv
CD: Cluster of Differentiation
CFC: colony forming cell
CLL: Chronic Lymphoid Leukemia
CNS: central nervous system
CRS: cytokine release syndrome
CTL: cytotoxic T lymphocyte
dp: double-positive
DRC: dose-response curve
EC_50_: Effective Concentration, at which 50% of targets are killed
INT: iodonitrotetrazolium chloride
LIC: leukemia initiating cells
LSC: leukemia stem cell
MNC: mononuclear cell
MPAL (NOS): Mixed Phenotype Acute Leukemia (No Other Specification)
n.d.: not determined
PB: peripheral blood
PBMC: peripheral blood mononuclear cells
PBS: phosphate-buffered saline
PHA: phytohemagglutinin
PFA: paraformaldehyde
RDL: redirected lysis
RLU: relative light units
scFv: single chain variable fragment
sp: single-positive
