# Supplementary material for: Dual-targeting triplebody 33-3-19 mediates selective lysis of biphenotypic CD19+ CD33+ leukemia cells
Source: Oncotarget. 2016 Mar 10;7(16):22579–89. doi: 10.18632/oncotarget.8022 (PMC5008383; doi:10.18632/oncotarget.8022)
Supplement: Supplementary file 1 [file oncotarget-07-22579-s001.pdf]

SUPPLEMENTARY FIGURES

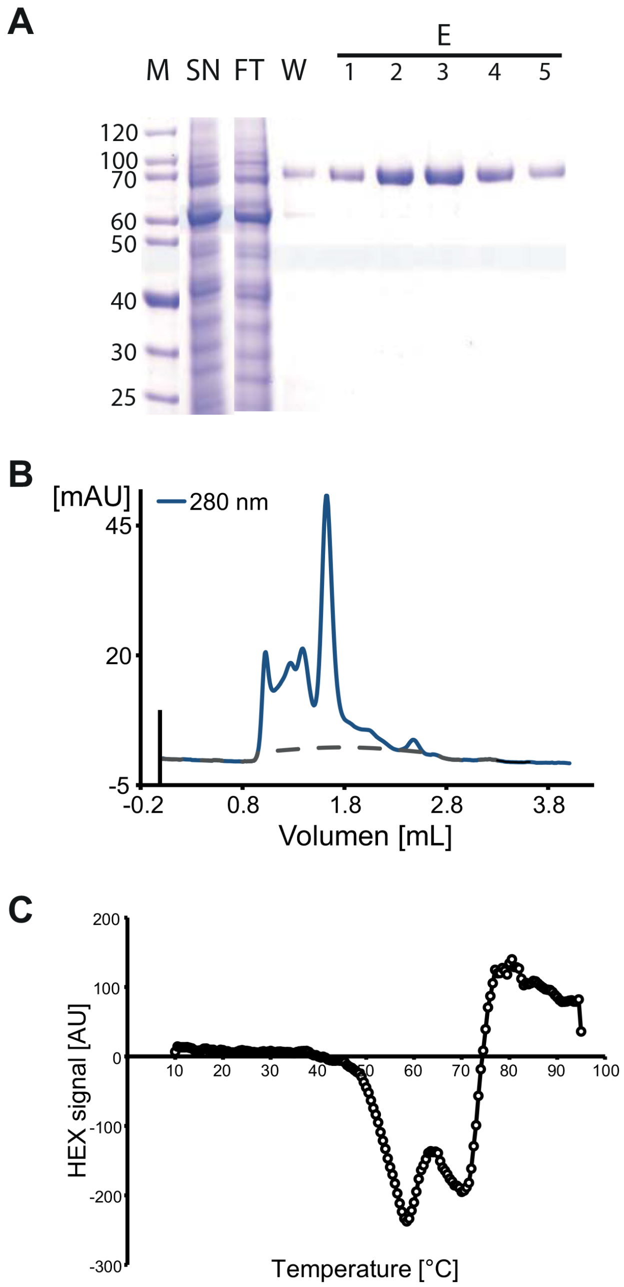

Supplementary Figure S1: Protein-chemical properties of dual-targeting T cell-engaging triplebody 33-3-19. A. SDS-polyacrylamide gel electrophoresis of protein fractions post Ni-NTA enrichment of triplebody 33-3-19 from the supernatant of a stable Freestyle 293F production cell pool (yield = 0.5 – 1.5 mg/L supernatant). M = protein marker, SN = supernatant, FT = flow through, W = wash fraction, E = elution fraction. **B.** Size exclusion chromatogram of triplebody 33-3-19. The monomer fraction (main peak) was collected and concentrated. Aliquots were flash-frozen in liquid nitrogen and stored at -80 °C until use (max. 2 months). **C.** Melting curve of 33-3-19 monomer as determined by a thermal shift assay. Melting point 1 (CD19 and CD3 scFvs) = 58.5°C; melting point 2 (CD33 scFv) = 70°C. Identification of the peaks from comparison of similar experiments performed earlier by our team with the triplebodies 33-16-123 (SPM-2) (N.C. Fenn, unpublished data) permitting identification of the transition caused by the CD33-specific scFv component and 19-3-19 (C.C. Roskopf, unpublished data) permitting identification of the transitions attributed to the CD19- and CD3-specific scFv components.

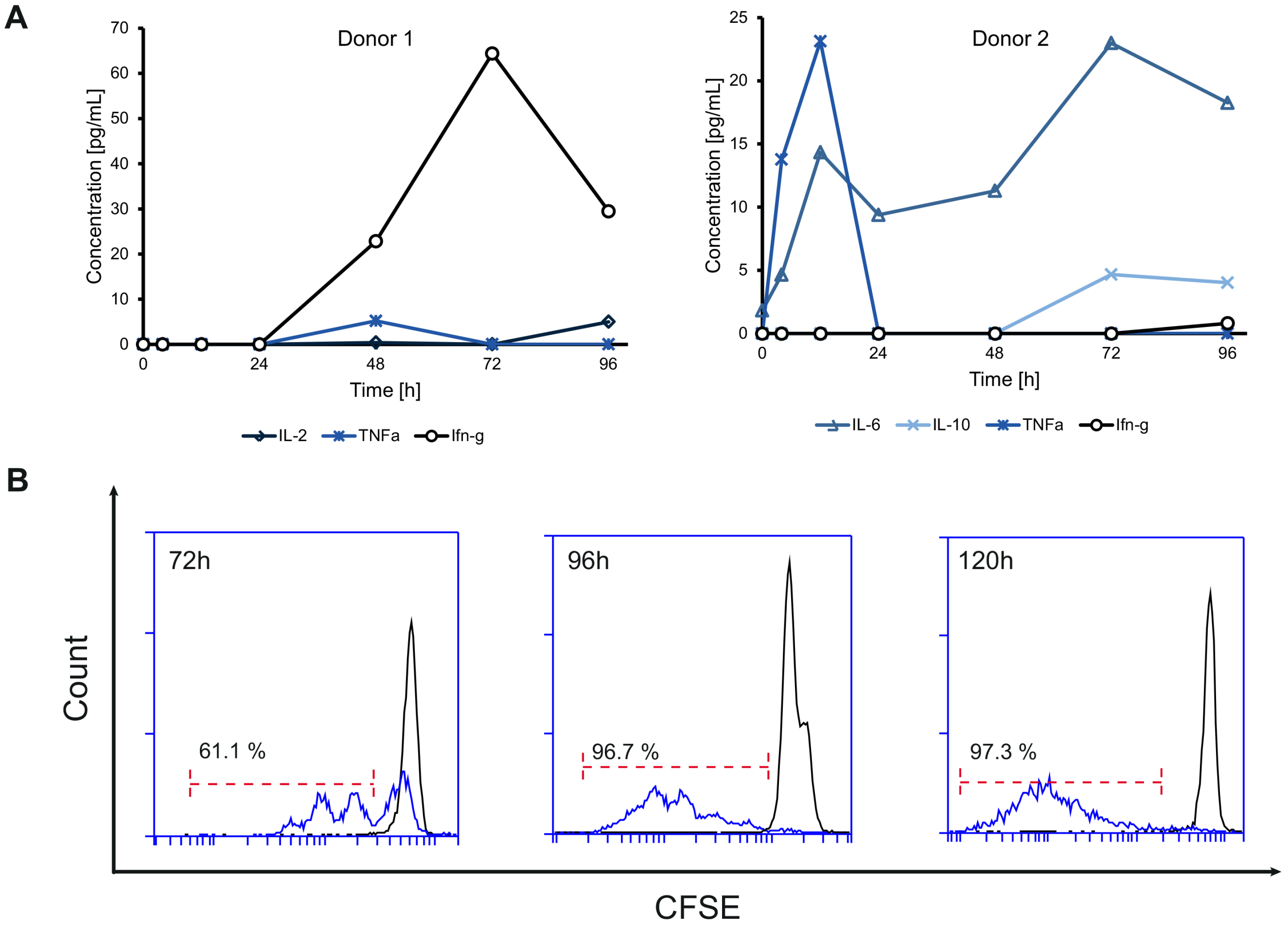

Supplementary Figure S2: T cell-activation induced by triplebody 33-3-19 and targets. Non-stimulated PBMCs from healthy unrelated donors were labelled with 5 µM CFSE and incubated with SEM target cells at an E : T ratio of 1 : 2 and a cell density of 3 * 10^5/ mL at t_0 (n = 3). **A.** After 0, 4, 12, 24, 48, 72 and 96 hours, cytokine levels in the medium were determined using the BD CBA™ Human Th1/Th2 Cytokine Kit II. Cytokine secretion profiles from 2 donors are shown. Both donors displayed complete target cell depletion and T cell proliferation was induced. Probably due to the very low cell numbers, cytokine concentrations increased slightly only, but IL-2, IL-6, IL-10, TNFα and IFN-γ were detectable, a similar profile to the one seen after treatment with the BiTE®s Blinatumomab and AMG330 [14, 15]. **B.** After 72, 96 and 120 hours T cell proliferation was assessed based on the dilution of the CellTrace™ CFSE proliferation dye.

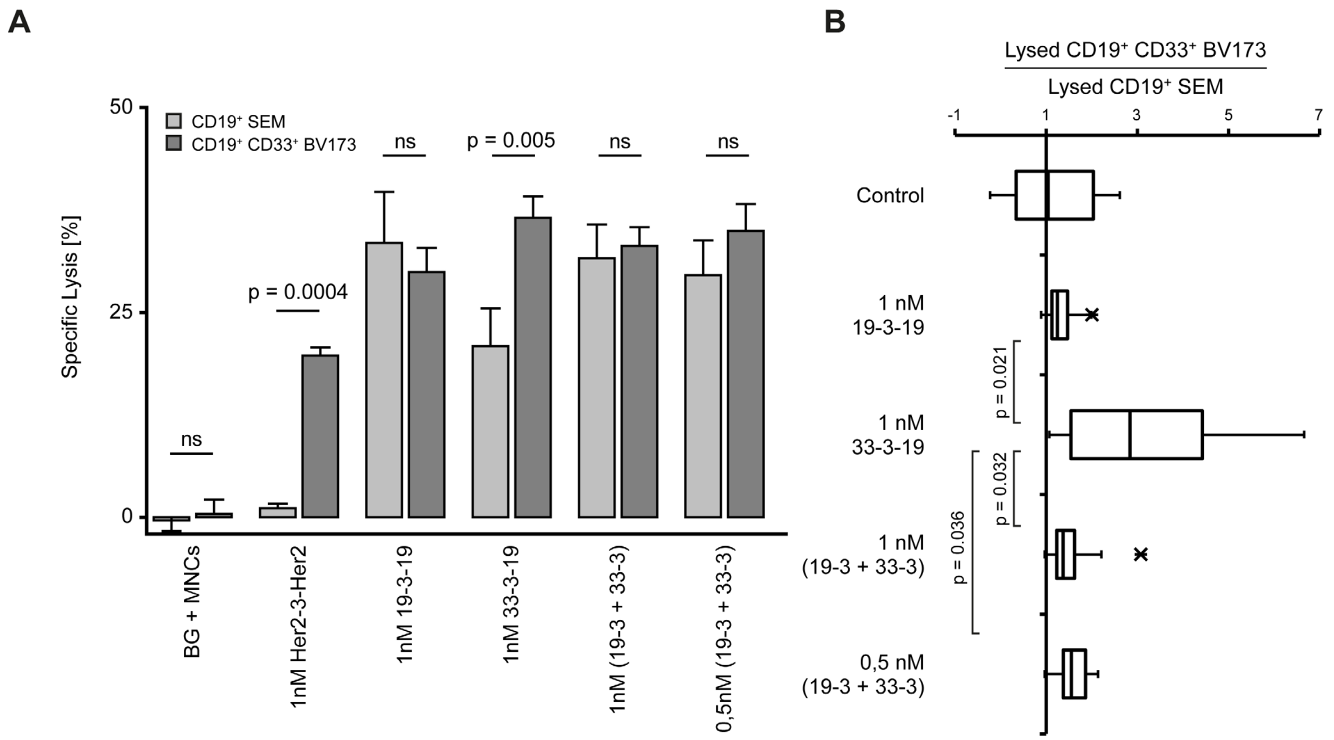

Supplementary Figure S3: Selective lysis of CD19+ CD33+ target cells at 1 nM 33-3-19. CD19 single-positive (sp) SEM target cells were Calcein-labelled in one and CD19/CD33 double-positive (dp) BV173 target cells were labelled in the other arm of a 3 hour Calcein release cytolysis assay with mixed target cell populations (E : sp T : dp T is 2 : 1 : 1; n = 11). **A.** Specific lysis of individual target cell populations in parallel reactions. In the presence of 1 nM dual-targeting triplebody 33-3-19, roughly twice as many BV173 target cells were lysed as SEM target cells (p = 0.0048). **B.** Box plot of the ratio of lysed dp-to-sp target cells, which was determined from the ratio of specific lysis achieved in each arm of the experiment, respectively.
